# Supplementary material for: Optimizing Protein Fiber Spinning to Develop Plant-Based Meat Analogs via Rheological and Physicochemical Analyses
Source: Foods. 2023 Aug 23;12(17):3161. doi: 10.3390/foods12173161 (PMC10487021; doi:10.3390/foods12173161)
Supplement: Supplementary file 1 [file foods-12-03161-s001.zip › foods-2570059-supplementary.pdf]

Supporting Information for

Utilization of fiber spinning extrusion to create fibrous plant-based meat analogs: Formulation, processing, and characterization

Kartik Joshi[1], Elnaz Shabani[1], Hualu Zhou[2], David Julian McClements[2], Jay H. Park[1*]

1. Department of Plastics Engineering, University of Massachusetts Lowell

2. Department of Food Science, University of Massachusetts Amherst

*Correspnding author: Jay_Park@uml.edu

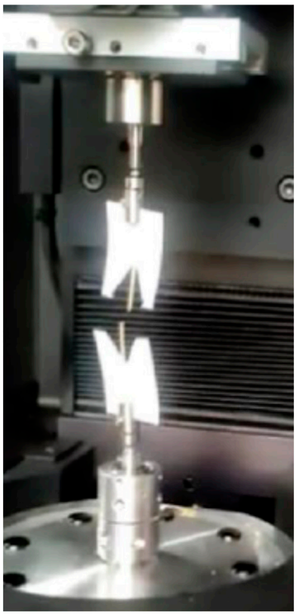

Figure S1. A representative tensile characterization of protein-based filaments using KLA T150 UTM.

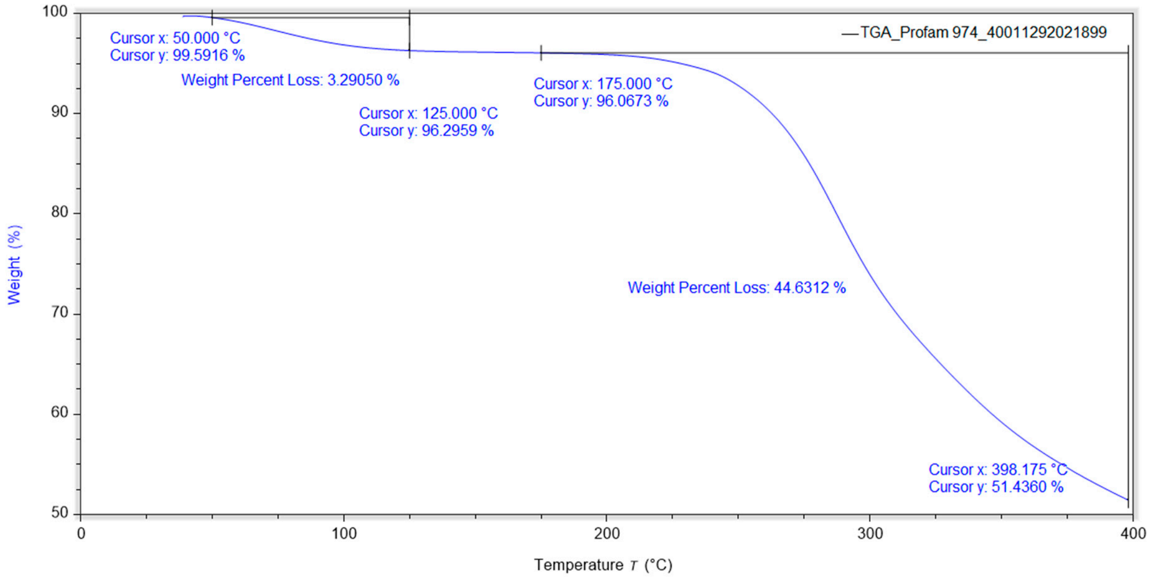

Figure S2. Thermogravimetric analysis (TGA) of Profam 974.

Table S1. TGA analysis experimental protocol

Segment 1	Initial Temperature 40.00 °C
Segment 2	Isothermal 1.00 min
Segment 3	Mark End of Cycle
Segment 4	Ramp 20.00 °C/min to 400.00 °C
Segment 5	Mark End of Cycle

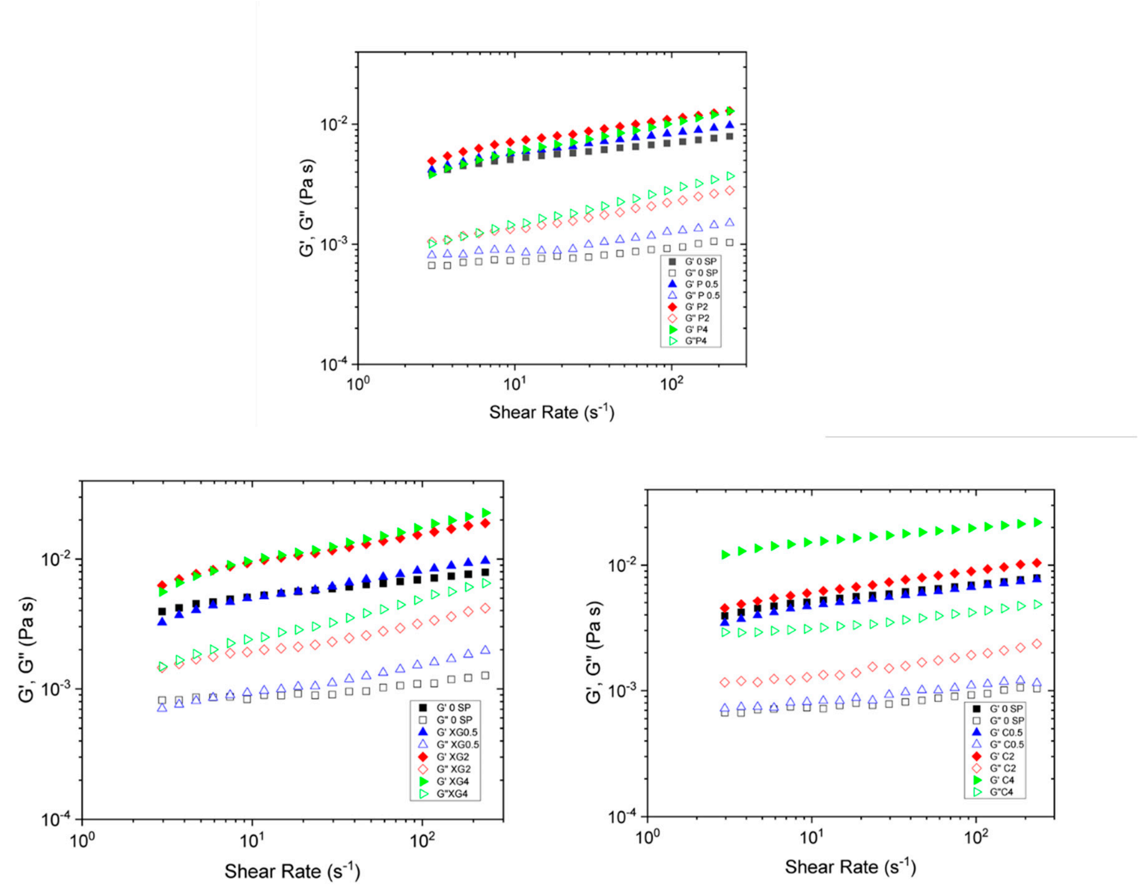

Figure S3. Storage and loss modulus with respect to shear rate are plotted for the formulations used in this study. The top plot shows results with varying concentrations of Pectin, while the bottom left shows those of Xanthan Gum, and the bottom right refers to Carrageenan.
